# Design Space Approach for the Optimization of Green Fluidized Bed Granulation Process in the Granulation of a Poorly Water-Soluble Fenofibrate Using Design of Experiment

**DOI:** 10.3390/pharmaceutics14071471

**Published:** 2022-07-15

**Authors:** Mohamed H. Fayed, Ahmed Alalaiwe, Ziyad S. Almalki, Doaa A. Helal

**Affiliations:** 1 Department of Pharmaceutics, Faculty of Pharmacy, Fayoum University, Fayoum 63514, Egypt; das12@fayoum.edu.eg; 2 Department of Pharmaceutics, College of Pharmacy, Prince Sattam Bin Abdulaziz University, Al-Kharj 11942, Saudi Arabia; a.alalaiwe@psau.edu.sa; 3 Department of Clinical Pharmacy, College of Pharmacy, Prince Sattam Bin Abdulaziz University, Al-Kharj 11942, Saudi Arabia; z.almalki@psau.edu.sa

**Keywords:** green fluidized bed granulation, design space, quality-by-design, design-of-experiment, granules and tablets

## Abstract

In the pharmaceutical industry, the systematic optimization of process variables using a quality-by-design (QbD) approach is highly precise, economic and ensures product quality. The current research presents the implementation of a design-of-experiment (DoE) driven QbD approach for the optimization of key process variables of the green fluidized bed granulation (GFBG) process. A 3^2^ full-factorial design was performed to explore the effect of water amount (X_1_; 1–6% *w*/*w*) and spray rate (X_2_; 2–8 g/min) as key process variables on critical quality attributes (CQAs) of granules and tablets. Regression analysis have demonstrated that changing the levels of X_1_ and X_2_ significantly affect (*p* ≤ 0.05) the CQAs of granules and tablets. Particularly, X_1_ was found to have the pronounced effect on the CQAs. The GFBG process was optimized, and a design space (DS) was built using numerical optimization. It was found that X_1_ and X_2_ at high (5.69% *w*/*w*) and low (2 g/min) levels, respectively, demonstrated the optimum operating conditions. By optimizing X_1_ and X_2_, GFBG could enhance the disintegration and dissolution of tablets containing a poorly water-soluble drug. The prediction error values of dependent responses were less than 5% that confirm validity, robustness and accuracy of the generated DS in optimization of GFBG.

## 1. Introduction

More than 70% of drugs are administered as solid dosage forms, such as tablets, which implies the extensive use of this tablet dosage form in healthcare sectors [1]. Prior to the tableting process, it is necessary to improve the flow properties of the materials so that the press can be evenly filled, resulting in tablets with uniform weight that can be manufactured [2]. This can be accomplished through the granulation process, which can be divided into two types: dry and wet granulation, depending on whether or not a liquid binder is utilized [3].

Wet granulation is a key unit process in the manufacture of solid dosage forms, particularly tablets and capsules [4]. The main goals of the granulation process are to enhance powder flow, increase material density, reducing segregation, improve content uniformity and enhance the tablets’ mechanical strength [5]. High-shear wet granulation (HSWG), twin-screw granulation and fluid-bed granulation (FBG) are the most common wet granulation technologies [6].

The FBG is one of the most widely used granulation technologies [7]. It converts poor flow powders to free-flowing granules, providing a single-step alternative to separate HSWG and fluid bed drying processes [8]. In addition, it produces porous granules, resulting in highly compactible granules and rapidly disintegrating tablets. The FBG is promising with respect to switching towards continuous processing. However, in the FBG, the drying process results in the granules’ attrition, an undesired size reduction phenomenon that results in the formation of fines, which is a quality-degrading side effect. The deteriorating effects of attrition on powder flow and granules homogeneity are previously reported [9,10,11].

To prevent undesirable attrition during the fluid-bed granulation/drying process, green fluidized-bed granulation (GFBG) is a possible alternative technique. The GFBG applies the concept of a moisture-activated dry granulation process, and mostly consists of two stages: granulation and moisture absorption [12,13]. During the granulation stage, drug, water-soluble diluents and binder are agglomerated by a little amount of water (1–4% *w*/*w*). In the absorption stage, the moisture available in the granules is absorbed by the addition of water-insoluble excipients, enabling the drying step to be omitted [14]. On the other hand, the GFBG technique does not involve a high amount of water, which helps to avoid issues, such as hydrolysis and degradation. Therefore, the GFBG technique may be a viable choice in terms of product quality and stability. However, the GFBG process needs more investigation, as it has the potential to avoid the issues face by conventional FBG processes.

Understanding the influence of process variables on granules’ and tablets’ attributes is critical for optimizing a process and increasing manufacturing efficiency [4]. In the pharmaceutical industry, the quality-by-design (QbD) approach has received more attention as a technique for developing products using a science and risk-based approach [15]. Implementation of QbD involves defining the critical process parameters (CPPs) that have a significant effect on the critical quality attributes (CQAs) of products. The design-of-experiment (DoE) approach has an important role in exploring the effects of large numbers of CPPs on CQAs of the product [16]. DoE aids in the development of high-quality product by decreasing the number of costly and time-consuming experiments [16]. The combinatorial effects of key process variables of GFBG on the CQAs of granules and tablets have largely been unexplored. Therefore, the main objective of the present study is to investigate the influence of CPPs of GFBG on CQAs of granules and corresponding tablets using a DoE approach in order to understand the influence of independent process variables on product quality and the implementation of effective control. Further, the GFBG process was optimized through the development of the process design space to produce desirable granules and tablets. In the present research, Fenofibrate was used as a model drug. It is categorized as a class II drug on the Biopharmaceutical Classification System, with a poor water solubility of <0.1 μg/mL [17]. It is widely used for the treatment of hyperlipidemia by reducing the blood levels of cholesterol and triglyceride [18].

## 2. Materials and Methods

### 2.1. Materials 

Fenofibrate was kindly donated from JPI Co. (Riyadh, Saudi Arabia). Polyvinylpyrrolidone, Kollidon^®^ 12 and D-α-Tocopherol polyethylene glycol 1000 succinate, and Kolliphor^®^ TPGS were obtained as a gift sample from BASF Co. (Ludwigshafen, Germany). Microcrystalline cellulose and Avicel^®^ PH-102 were gained as a gift sample from DuPont Nutrition USA, Inc. (Wilmington, NC, USA). Lactose monohydrate and Granulac 200^®^ were obtained from Meggle (Wasserburg, Germany). Colloidal silicon dioxide and Aerosil 200^®^ were purchased from Evonik (Hanau-Wolfgang, Germany). Sodium stearyl fumarate, Pruv^®^ and sodium starch glycolate, and Explotab^®^ were obtained as a gift sample form JRS pharma (Rosenberg, Germany). Sodium dihydrogen phosphate dehydrate, disodium hydrogen phosphate dodecahydrate and sodium hydroxide were purchased from Merck (Darmstadt, Germany).

### 2.2. Design of Experiments 

In the present study, two key process variables, namely water amount (X_1_, 1–6% *w*/*w*) and spray rate (X_2_, 2–8 g/min), were investigated to understand their effects on CQAs of prepared granules and tablets using a 3^2^ full-factorial design. According to the results of the preliminary experiments, the low, moderate and high levels of each independent variable were chosen. The independent process variables and their levels are listed in Table 1. For a 3^2^ full-factorial design, nine runs were generated as shown in Table 2. The experimental design and data analysis were carried out using Design-Expert software (version 11, State-ease, Inc., Minneapolis, MN, USA). The following polynomial equation was utilized to fit the experimental data of all granule and tablet properties Equation (1):

(1) $$Y=b_{0}+b_{1}X_{1}+b_{2}X_{2}+b_{12}X_{1}X_{2}+b_{11}X_{1}{}^{2}+b_{22}X_{2}{}^{2}$$

where Y is a measured granule or tablet response associated with each variable level combination; b_0_ is an intercept; b_1_ and b_2_ are regression coefficients; and X_1_ and X_2_ are the coded levels of investigated variables. The terms of X_1_X_2_ and $X_{i}{}^{2}$ (i = 1 and 2) symbolize the interaction and quadratic terms, respectively. The generated design was validated by calculating the prediction error (PE) using Equation (2). A PE of less than 5% proves the validity of the experimental design [19].

(2) $$\mathrm{PE}=\left(\;\frac{\mathrm{Predicted}\;\mathrm{value}-\mathrm{Experimental}\;\mathrm{value}\;}{\mathrm{Predicted}\;\mathrm{value}}\right)\times 100$$

### 2.3. Green Fluidized Bed Granulation

Table 3 shows the formulation used in the present study. The granulation process was conducted in a fluidized bed granulator (BOSCH Packaging Technology, Schopfheim, Germany) at a 700 g scale for all trials. During the entire process, the velocity of fluidizing air was 0.3–0.4 m^3^/min without the use of heater. The drug, lactose monohydrate, polyvinylpyrolidone and TPGS were initially mixed (2 min) and granulated by spraying water (1–6% *w*/*w*) using a centered top spray nozzle (spray rate 2–8 g/min). For the 5 min absorption stage, colloidal silicon dioxide and microcrystalline cellulose were added as moisture absorbents. Finally, the disintegrant “sodium starch glycolate” and lubricant “sodium stearyl fumarate” were added directly into the granulator and mixed for 1.5 min and 2 min, respectively.

### 2.4. Tablet Compaction

The lubricated granules were used for tablet compaction on an automated single-punch tablet press (Erweka EP-1, Apparatebau, Langen, Germany) using 12 mm round flat-faced tooling at 12 kN. The target tablet weight was 500 mg. The machine was adjusted to produce approximately 50–60 tablets per minute. The produced tablets were collected, stored and evaluated.

### 2.5. Granules Evaluation

#### 2.5.1. Particle Size Distribution (PSD)

The granules (*n* = 3) were analyzed for PSD by the laser diffraction method as described in our previous work [20]. Fines were considered as granules with a diameter smaller than 50 µm.

#### 2.5.2. Bulk Density 

Granule samples of 15 g (*n* = 3) were carefully fed into a 50 mL graduated cylinder. The granules weight (m) and volume (v) were determined, and bulk density (ρ) was calculated using Equation (3).

(3) Bulk Density= m/v

#### 2.5.3. Granules Flow

A static angle of repose (AoR) method was used to evaluate the flowability of prepared granules as well as the physical mixture. The powder (*n* = 3) was carefully poured using a dry conical funnel fixed at a constant height (H). The base of the cone’s diameter (D) was determined, and the AoR was calculated using Equation (4).

(4) tan(α)=2H/D

### 2.6. Tablets Evaluation

#### 2.6.1. Tablet Weight and Tensile Strength

The tablet weight, thickness (t), diameter (d) and breaking force (f) were measured (*n* = 10) by Erweka Multi-Check 5.1 (ERWEKA, Heusenstamm, Germany). The tensile strength (σ) of the prepared tablet was calculated using Equation (5).

(5) σ =2F/πtd

#### 2.6.2. Tablet Friability

Tablet friability was detected by the feeding of 10 tablets in the drum of friability tester (ERWEKA, Heusenstamm, Germany); rotating at 25 rpm for 4 min. Tablet friability was measured using Equation (6).

(6) $$\mathrm{Friability}\;\left(\%\right)=\left[\left(W_{1}-W_{2}\right)/W_{1}\right]\times 100$$

where W_1_ is tablets weight before the test and W_2_ is the tablets’ weight after the test. 

#### 2.6.3. Tablet Disintegration Time (DT)

The DT was estimated (*n* = 6) utilizing the automatic disintegration apparatus (ERWEKA, Heusenstamm, Germany). The distilled water at 37 ± 0.5 °C was used as the disintegration medium. The DT was determined using a digital stopwatch. The DT was achieved when the tablets were fully disintegrated, and no lumps remained in the disintegration medium.

#### 2.6.4. Tablet Dissolution

Tablet dissolution (*n* = 6) was conducted using an USP apparatus type II (ERWEKA, Heusenstamm, Germany) maintained at 37 ± 0.5 °C and 100 RPM. The test was performed in 900 mL of phosphate buffer (1.73 g L^−1^ of sodium dihydrogen phosphate dihydrate and 4.92 g L^−1^ disodium hydrogen phosphate dodecahydrate in highly purified water, pH 6.8), to simulate intestinal fluid, containing 0.75% sodium lauryl sulphate to achieve sink condition. Aliquots of 3 mL were collected at predetermined time intervals (5, 10, 15, 30, 45 and 60 min) and an equivalent volume of fresh medium was replenished at each time point. The withdrawn samples were filtered through a 0.45 µm membrane filter. The filtrate was centrifuged at 11,000 rpm for 5 min and the UV absorbance at 289 nm was used to estimate the fenofibrate content using a UV–vis spectrophotometer (Shimadzu, UV-1800, Kyoto, Japan) [21]. Dissolution profiles were compared using similarity facto (f_2_), which is defined by Equation (7) [22].

(7) $$f_{2}=50\log\left\{{\left[1+1/n\sum^{\;}n_{t=1}{\left(R_{t}-T_{t}\right)}^{2}\right]}^{-0.5}\times 100\right\}$$

where n is the number of dissolution sampling times, and R_t_ and T_t_ are the percentage of drug released at each time point for the reference and test, respectively. An f_2_ value of more than 50 indicates that the two dissolution profiles are similar.

## 3. Results and Discussion

### 3.1. Data Fitting

**Figure 1:**
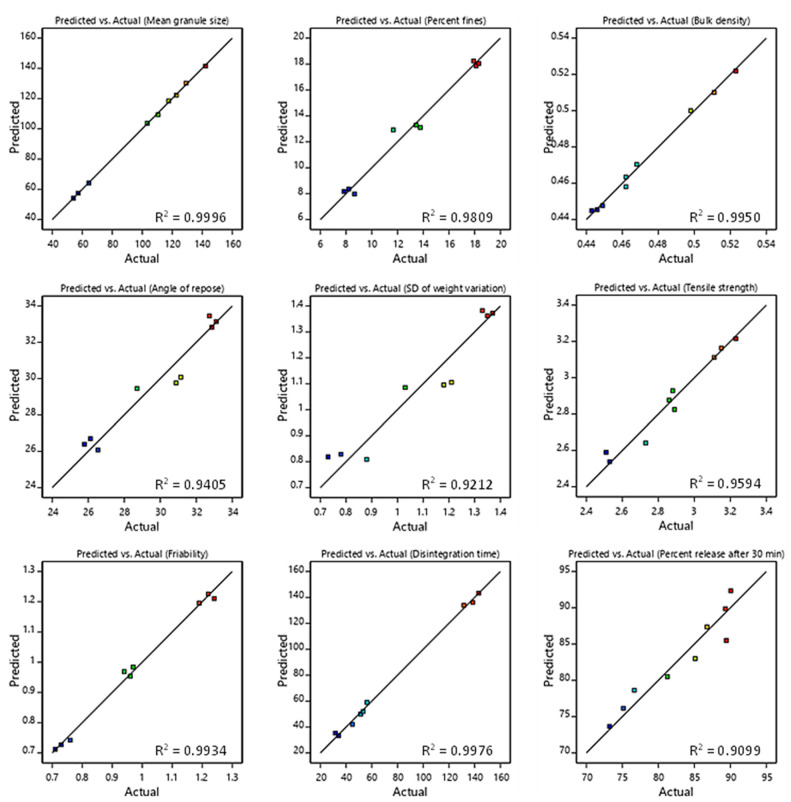
Linear correlation plot relating; mean granule size, percent fines, bulk density, angle of repose, SD of weight variation, tensile strength, friability, disintegration time and drug release after 30 min, between the predicted and the actual values.

The model fitting results including the coefficient of determination (R^2^), adjusted R^2^, predicted R^2^, associated probability (*p*-value) and adequate precision are summarized in Table 4. All the suggested models have R^2^ > 0.9, which indicates a high significance and high fitness data to the models. Additionally, the p-value for all suggested models was less than 0.05 at a 5.0 percent significance level, indicating that the models were well-fit to the data. Furthermore, the predicted R^2^ agrees reasonably well with the adjusted R^2^ (i.e., the difference is less than 0.2), which demonstrates good data fitting. As shown in Figure 1, a linear correlation between experimental results and results predicted by the model, suggests a good model fit. For all suggested models, adequate precision was more than 4.0. This indicated that the suggested models could be utilized to navigate the design space.

### 3.2. Influence of Process Variables on Granule Properties

#### 3.2.1. Mean Granule Size (y_1_) and Percent Fines (y_2_)

As depicted in Table 5, the mean granule size (D_50_) ranged from 54.11 ± 0.25 to 142.13 ± 0.35. Compared with the mean size of physical mixture, it can be seen that granule size was significantly increased with the increasing water amount from 1–6% *w*/*w* and spray rate from 2–8 g/min. The influence of X_1_ and X_2_ on D_50_ was demonstrated by the following polynomial Equation (8).

(8) $$D_{50}\left(\mu m\right)=109.38+36.37{\;X}_{1}+7.33X_{2}+2.28{\;X}_{1}{\;X}_{2}-15.53{\;X}_{1}^{2}+1.7{\;X}_{2}^{2}$$

**Figure 2:**
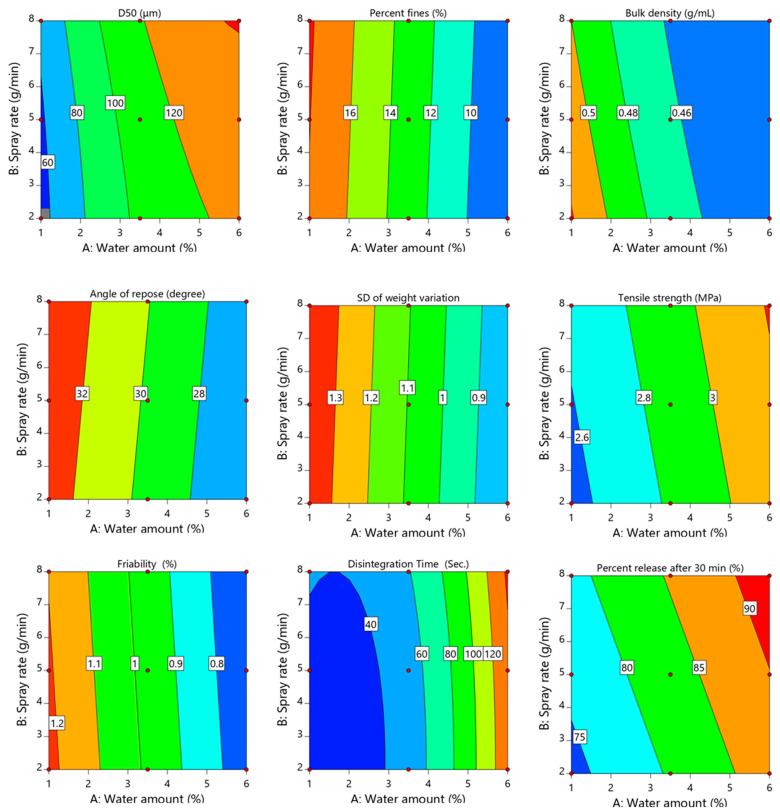
Contour plots showing the effect of water amount (X_1_) and spray rate (X_2_) on mean granule size (y_1_), percent fines (y_2_), bulk density (y_3_), angle of repose (y_4_), SD of weight variation (y_5_), tensile strength (y_6_), friability (y_7_), disintegration time (y_8_) and percent release after 30 min (y_9_).

Regression analysis (Table 6) demonstrated that all process variables had significant effects on D_50_ (*p* < 0.0001 for amount of water and *p* = 0.0005 for spray rate). However, the sum of square values suggested that the amount of water was the most significant process variable (7935.93 for water amount and 322.52 for spray rate). All the significant parameters had a positive influence as indicated by the positive sign of the regression coefficient (+36.37 for water amount and +7.33 for spray rate), indicating that the increasing level of these variables results in a larger granule size. Similarly, the interaction terms X_1_X_2_ (*p* = 0.026) and the quadratic term X_1_^2^ (*p* = 0.0003) increase the mean granule size. Combined with the contour plot (Figure 2), it is clear that D_50_ attained its maximum value with the concurrent increase in the investigated process variables (X_1_ and X_2_). This could be due to the fact that water induced the powder to stick and coalesce more easily [20,23]. In addition, increasing the spray rate results in more availability of water in the spraying zone and, hence, more wetting and sticking of the particles [23]. This result is in agreement with previously reported work [23,24].

As shown in Table 5, the percent fines ranged from 7.84 ± 0.028 to 18.32 ± 0.052 %. The impact of the process parameters on percent fines was described by the following first-order polynomial Equation (9).

(9) $$\mathrm{Percent}\;\mathrm{fines}\;\left(\%\right)=13.11–4.94{\;X}_{1}+0.19{\;X}_{2}$$

Regression analysis (Table 6) demonstrates that the amount of fines was mainly influenced by water amount (*p* < 0.0001), while spray rate had no significant effect (*p* = 0.5248). According to the negative sign of the regression coefficient (−4.94 for water amount), the significant term had a negative effect, indicating that as the amount of water increased, the amount of fines decreased. This could be attributed to an inadequate amount of water, which results in weak and fragile granules with a high percentage of fines [25]. 

#### 3.2.2. Bulk Density (y_3_)

The bulk density of produced granules ranged from 0.443 ± 0.009 to 0.523 ± 0.016 g/mL, as indicated in Table 5. The following polynomial equation was used to demonstrate the impact of process parameters on the granule density Equation (10).

(10) $$\mathrm{Bulk}\;\mathrm{density}\;\left(g/\mathrm{Ml}\right)=0.4634\;–0.0323{\;X}_{1}–0.0062{\;X}_{2}+0.0047{\;X}_{1}{\;X}_{2}+0.0143{\;X}_{1}^{2}+0.0008{\;X}_{2}^{2}$$

Regression analysis (Table 6) indicated that all process variables had a significant negative effect (*p* = 0.0002 for amount of water and *p* = 0.0214 for spray rate) on the bulk density of granules with respect to the negative sign of the regression coefficient (−0.0323 for the amount of water and −0.0062 for the spray rate). However, the amount of water had the predominant effect based on the sum of square values (0.0063 for water amount and 0.0002 for spray rate). Combined with the contour plot shown in Figure 2, the granule bulk density decreased with increasing water amount. The fluid bed produces small granules that have a low free-fall velocity and a high surface-to-mass ratio; this results in a lower bulk density of prepared granules (25). The two-way interaction between the investigated factors had no significant effect (*p* = 0.0686) on granules bulk density.

#### 3.2.3. Flowability (y_4_)

The flow of powder during die filling, one of the crucial steps in the manufacturing of tablets, can affect not only the overall efficiency of the tableting process, but also the final product quality, such as weight and content uniformity [26,27]. The flowability of powders used in the manufacturing of tablets has a significant impact on the uniformity of tablet weight and drug content [28]. The angle of repose is an important test for determining the flowability of powders and granules [19]. Its value below 35 indicates good powder flowability, while a value above 35 indicates poor flowability [29]. Comparing the flowability of the physical mixture (the angle of repose of the physical mixture = 35.76 ± 0.311) with the flowability of granules prepared by GFBG, it can be seen that the flowability of granules prepared under various process variables ranged from 26.12 ± 0.295 to 33.11 ± 0.523° (Table 5). This indicates that the flowability of the granules obtained by the GFBG process were significantly improved compared to flowability of the physical mixture. The first-order polynomial equation generated by regression analysis of granules angle of repose is described by Equation (11).

(11) $$\mathrm{Angle}\;\mathrm{of}\;\mathrm{repose}\;\left({}^{\circ}\right)=29.77\;–\;3.38{\;X}_{1}+0.3117{\;X}_{2}$$

The regression analysis (Table 6) indicates that the amount of water had a significant effect on granules’ flowability (*p* < 0.0001 for amount of water and *p* = 0.4050 for spray rate). According to the polynomial equation, the negative sign of regression coefficients (−3.38) showed that the water amount has an inverse relationship with the granule angle of repose. Combined with the contour plot (Figure 2), it is clear that angle of repose reaches its minimum value with the concurrent increase in water amount, as displayed on the right side of the contour plot. This indicates that, the granules produced using green fluid bed granulation with spraying of a water amount of more than 3.5% has better flowability because of uniform and high granule size. It is reported that increasing water amount improves granules flowability [14].

### 3.3. Influence of Process Variables on Tablet Properties

#### 3.3.1. Weight Variation

The tablet weight ranged from 497.56 ± 1.35 to 501.71 ± 1.33 mg (Table 7). Based on the USP criteria for tablet weight variation, all prepared tablets achieved an acceptable weight variation with an SD of less than 2. This indicates that the prepared granules demonstrated an acceptable flow and bulk density [30]. The empirical model of first-order polynomial Equation (12) described the impact of the process parameters on weight variation of prepared tablets.

(12) $$\mathrm{SD}\;\mathrm{of}\;\mathrm{weight}\;\mathrm{variation}\;=1.10\;–\;0.2767{\;X}_{1}+0.01{\;X}_{2}$$

As shown in Table 8, only the amount of water had a significant effect on variation of tablet weight (*p* = 0.0002 for amount of water and *p* = 0.7725 for spray rate). The regression coefficients in Equation (12) indicated that the amount of water had negative impact on tablet weight variation according to the negative sign of coefficient estimate (−0.2767). Combined with the contour plot (Figure 2) it is clear that tablet weight variation attained its minimum value with the simultaneous increase in the water amount. By contrast, weight variation was highest when water amount was at its minimum value. This result was consistent with the results of powder flow (Section 3.2.3).

#### 3.3.2. Tensile Strength and Friability

The tensile strength of the tablet is an important indicator to evaluate the mechanical strength of the obtained tablet [19]. As shown in Table 7, the tablet tensile strength ranged from 2.51 ± 0.13 to 3.23 ± 0.24 MPa. This indicates that granules obtained by the green fluidized bed granulation method could be compressed into tablets with acceptable mechanical strength. On the other hand, regression analysis (Table 8) showed that the amount of water had a significant impact (*p* < 0.0001) on tablet tensile strength. The empirical model of the first-order polynomial Equation (13) describes the effect of the process parameters on tablet tensile strength.

(13) $$\mathrm{Tensile}\;\mathrm{strength}\;\left(\mathrm{MPa}\right)=2.88+0.2867{\;X}_{1}+0.0517{\;X}_{2}$$

As shown in Equation (12), the positive coefficients of water amount (+0.2867) revealed that a higher value for this variable results in a higher tensile strength. Combined with the contour plot in Figure 2, it is concluded that increasing the water amount sprayed (up to 6%) could improve the tablet mechanical strength. It was reported that green fluidized bed granulation has the potential to increase the granules compaction properties by enhancing plastic deformability [13]. Additionally, the present granulation method produces looser and large surface area granules that contribute to the strong inter-particle bonding [14]. 

Friability of tablet is also an important index to evaluate the tablet mechanical strength [31]. As shown in Table 7, the friability of tablet ranged from 0.71 ± 0.01 to 1.24 ± 0.02 %. Runs 1, 2 and 3 (granulated with 2% water amount) produces friable tablets since the prepared tablets lose more than 1.0 % of weight after undergoing the friability test. This is due to inadequate wetting of powder particles and the generation of large amounts of fines (17–18 %) [20,32]. However, runs 4–9 (3.5–6 % water amount) produced acceptable tablets since the tablet weight loss was less than 1.0%. An ANOVA analysis (Table 8) revealed that the amount of water had a significant effect on tablet friability (*p* < 0.0001). The empirical model of first-order polynomial Equation (14) describes the impact of the process parameters on tablet tensile strength.

(14) $$\mathrm{Friability}\;\left(\%\right)=0.9689-0.2417{\;X}_{1}-0.0150{\;X}_{2}$$

As shown in Equation (13), the negative coefficient of water amount (−0.2417) demonstrates that a higher value for this variable results in a lower weight loss of tablet. Combined with the contour plot in Figure 2, it is concluded that increasing the water amount (more than 2%) could decrease the friability of the prepared tablets and provide acceptable tablet in term of the USP criteria.

#### 3.3.3. Disintegration Time

As shown in Table 7, disintegration time (DT) of the obtained tablet ranged from 31.69 ± 1.31 to 143.12 ± 0.86 s. It was observed that as the water amount increased, the DT was significantly increased. Regression analysis shown in Table 8 indicates that water amount had a significant effect (*p* < 0.0001) on the DT of the tablets. The empirical model of polynomial Equation (15) describes the influence of the process parameters on DT.

(15) $$\mathrm{DT}\;\left(s\right)=52.0+50.42{\;X}_{1}+4.51{\;X}_{2}+0.1975{\;X}_{1}{\;X}_{2}+0.3373{\;X}_{1}^{2}+2.45{\;X}_{2}^{2}$$

Based on the polynomial Equation (15), the positive coefficient of water amount (+50.42) demonstrates that a higher value for this variable results in longer tablet disintegration. On the other hand, the two-way interaction between the investigated variables had no significant (*p* = 0.9235) effect on tablet disintegration. The contour plot shown in Figure 2 shows the influence of water amount and spray rate on the DT. It is observed that tablet DT attained its minimum value with the simultaneous decrease in the water amount. The rapid disintegration may be due to the low tensile strength of tablets that prepared a low amount of water.

#### 3.3.4. In Vitro Drug Release

**Figure 3:**
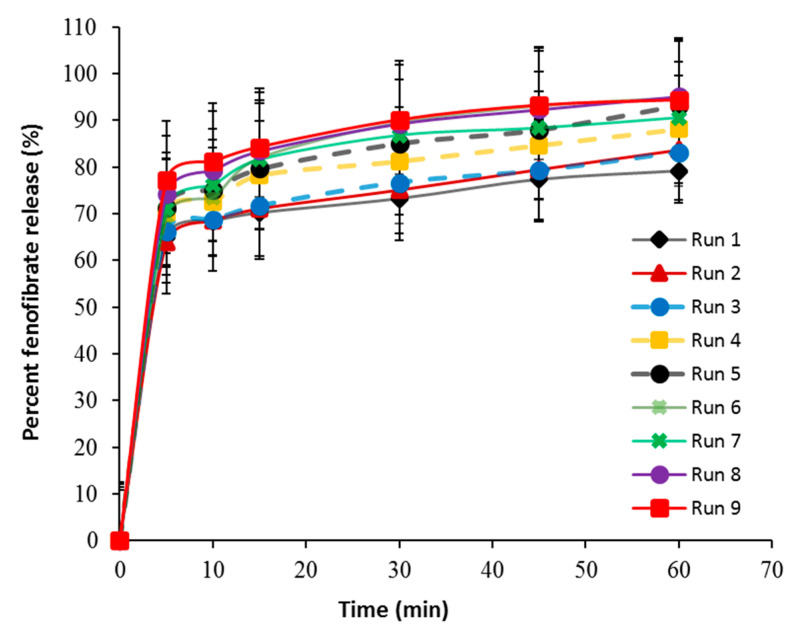
In vitro release profiles of fenofibrate tablets based on 3^2^ full-factorial Design.

Figure 3 shows the release profiles of fenofibrate at different process variables. As shown in Table 7 and Figure 3, only runs 5–9 demonstrate an acceptable drug release profile (i.e., 85 % release after 30 min), while runs 1, 2, 3 and 4 released 73.23 ± 3.15, 75.15 ± 1.14, 76.66 ± 2.13 and 81.25 ± 2.35 %, respectively, after 30 min. Regression analysis (Table 8) indicates that the water amount and spray rate had a significant effect (*p* = 0.0003 for the amount of water and *p* = 0.0376 for spray rate) on fenofibrate dissolution. However, water amount was the most significant factor with respect to the sum of square values (281.67 for water amount and 37.20 for spray rate). The following first-order polynomial equation was applied to describe the relationship between the independent process parameters and drug release Equation (16).

(16) $$\mathrm{Percent}\;\mathrm{drug}\;\mathrm{release}\;\mathrm{after}\;30\;\min\;\left(\%\right)=83.00+6.85{\;X}_{1}+2.49{\;X}_{2}$$

According to the polynomial Equation (16), the positive sign of regression coefficients (+6.85 for water amount and +2.49 for spray rate) shows that water amount and spray rate had a positive effect on fenofibrate release. Combined with the contour plot (Figure 2), it is clear that drug release attained its maximum value with the concurrent increase in the investigated process variables as exhibited in the upper right corner of the contour plot. The result suggests that dissolution of fenofibrate from prepared tablets can potentially be improved by increasing the water amount and spray rate. This could be attributed to high levels of water amount and spray rate produced by low-density granules, as previously described (Section 3.2.2), which rapidly eroded and rapidly released the drug. This resulted in the rapid release and maximum concentration of fenofibrate in dissolution medium. It was reported that granulation using fluid-bed results in the formation of low density and high porous granules that rapidly erode [31,33]. On the other hand, incorporation of TPGS into the granulation process could improves the dissolution profile of fenofibrate. TPGS was found to improve the dissolution and apparent solubility of poorly water-soluble drugs in the granulation process [34]. As shown in Table 9, the comparison of the obtained dissolution profiles clearly revealed a significant increase in the dissolution rates upon increasing the water amount from 1 to 6% *w*/*w* (as seen in run 1 # 7, run 2 # 8 and run 3 # 9, and the dissolution profile was dissimilar since f_2_ was less than 50). The results of f_2_ similarity confirm the results of regression analysis. 

### 3.4. Process Optimization, Design Space and Validation

**Figure 4:**
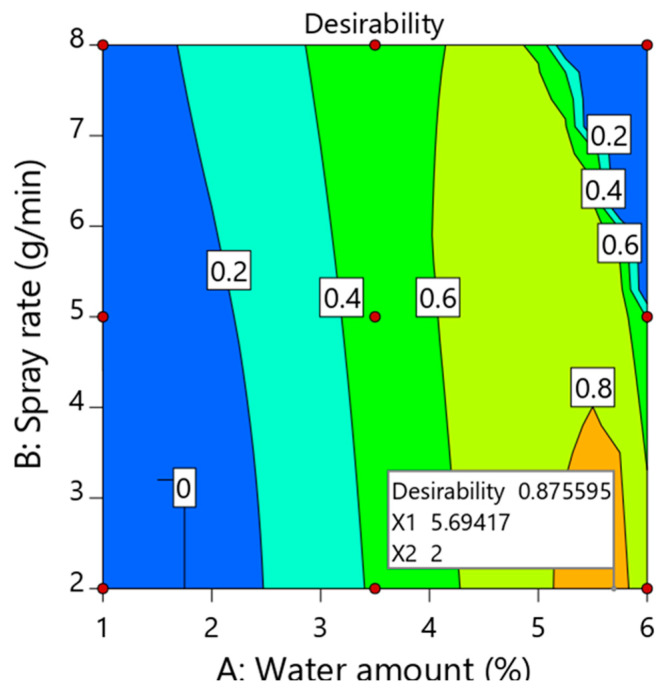
Optimization plot showing the influence of water amount (X_1_) and spray rate (X_2_) on overall desirability.

The objective of the optimization step is to define the optimum level of each independent variable, in order to produce a product of desired quality attributes [35]. The process parameters should be operated within a defined design space (DS) to obtain the desired quality attributes [15]. Optimization of investigated key process variables was carried out using a numerical optimization method. As shown in Table 10, values of SD of weight variation, tensile strength, friability, DT and percent release within 30 min were set to the target of 0.8, 3 MPa, 0.75% 120 sec and 85%, respectively, to generate the optimized process conditions using desirability function. According to the set criteria, water amount and spray rate at high (5.69% *w*/*w*) and low levels (2 g/min), respectively, demonstrate the optimized conditions with a desirability value close to one (Figure 4). As shown in Table 11, granulation at optimized conditions results in tablets with an SD of weight variation of 0.82, tensile strength of 3.20 ± 0.46, friability of 0.74 ± 0.05, DT of 125.12 ± 2.43 and percent release of 88.21 ± 3.31 after 30 min. In addition, the prediction error between the experimental value and the corresponding predicted value was less than 5.0%, which was acceptable. This revealed that the model was valid with reliable and effective predictive power [36].

## 4. Conclusions

In the present study, the DoE approach as a part of the QbD paradigm was used to optimize the GFBG process. A 3^2^ full-factorial design was performed to explore the effect of water amount (1–6% *w*/*w*) and spray rate (2–8 g/min) as key process variables of GFBG on CQAs of granules (D_50_, percent fines, bulk density and flowability) and tablets (weight variation, mechanical strength, DT and drug release). Regression analysis demonstrated that the investigated process variables have significant effect (*p* ≤ 0.05) on CQAs of granules and corresponding tablets. Particularly, water amount was found to have the more pronounced effect on granules’ and tablets’ properties. The results reveal that the desired quality attributes of granules and tablets could be optimized by adjusting the process variables of water amount and spray rate during the GFBG process. A design space was developed based on tensile strength, disintegration and dissolution of prepared tablets using desirability function. The results of the prediction error test have shown the validity, reliability and effectiveness of the generated design space in optimizing the GFBG process. By optimizing the water amount and spray rate, GFBG could improve the disintegration and dissolution of tablets containing a poorly water-soluble drug. Compared with the conventional fluid-bed granulation process, GFBG was found to be simple, cost-effective and a less energy consuming process, yielding granules and tablets with desirable quality attributes as it requires no heating step. Thus, this granulation process is a promising method for the granulation of moisture and heat sensitive materials. In summary, the current study increases knowledge of the impact of the GFBG process parameters on properties of granules and tablets, and it might be used to construct a mechanistic model for the granulation process. 

## Figures and Tables

**Table 1:** Key process variables used in the design of experiments.

Coded Levels	Water Amount (% *w*/*w*)	Spray Rate (g/min)
−1	1	2
0	3.5	5
1	6	8

−1: factor at low level; 0: factor at medium level; 1: factor at high level.

**Table 2:** A 3^2^ full-factorial experimental design.

Run	Water Amount (% *w*/*w*)	Spray Rate (g/min)
1	1.0	2
2	1.0	5
3	1.0	8
4	3.5	2
5	3.5	5
6	3.5	8
7	6	2
8	6	5
9	6	8

**Table 3:** Formulation used in the granulation experiments.

Ingredients	% *w*/*w*	Function
Fenofibrate	32	Poorly water-soluble drug
Lactose monohydrate	46.5	Water soluble filler
Microcrystalline cellulose PH-102	10	Moisture absorbents
Polyvinylpyrrolidone K12	5	Binder
D-α-Tocopherol polyethylene glycol 1000 succinate	2	Non-ionic surfactant
Sodium starch glycolate	2	Super-disintegrant
Colloidal silicone dioxide	1.5	Moisture absorbents
Sodium stearyl fumarate	1	Hydrophilic lubricant
Total	100	-

**Table 4:** Summary of model fitting and statistical analysis.

Responses	Suggested Model	*p*-Value	R2	Adjusted R2	Predicted R2	Adequate Precision
Y1:D50	Quadratic	<0.0001	0.9996	0.9989	0.9965	96.642
Y2: Percent fines	Linear	<0.0001	0.9809	0.9746	0.9596	25.775
Y3: Bulk density	Quadratic	0.0012	0.9950	0.9868	0.9428	27.664
Y4: Angle of repose	Linear	0.0002	0.9405	0.9207	0.8776	14.986
Y5: SD of weight variation	Linear	0.0005	0.9212	0.8949	0.8308	12.261
Y6: Tensile strength	Linear	<0.0001	0.9594	0.9459	0.9024	19.571
Y7: Friability	Linear	<0.0001	0.9934	0.9913	0.9875	45.194
Y8: Disintegration time	Quadratic	0.0004	0.9976	0.9935	0.9713	35.529
Y9: Percent release after 30 min	Linear	0.0014	0.9099	0.8798	0.7992	14.105

**Table 5:** Measured response results of physical mixture and prepared granules (mean ± SD).

Formula	D_10_ (µm ± SD )	D_50_ (µm ± SD )	D_90_ (µm ± SD )	Percent Fines (% ± SD )	Bulk Density (g/mL ± SD)	AoR (Degree ± SD)
Physical Mixture	12.22 ± 0.31	51.15 ± 0.22	107.36 ± 0.33	21.13 ± 0.022	0.411 ± 0.031	35.76 ± 0.311
1	14.31 ± 0.29	54.11 ± 0.25	114.25 ± 0.24	18.11 ± 0.034	0.523 ± 0.016	32.87 ± 0.331
2	17.12 ± 0.31	57.32 ± 0.34	116.43 ± 0.36	18.32 ± 0.052	0.511 ± 0.004	33.11 ± 0.523
3	19.02 ± 0.33	64.42 ± 0.31	123.11 ± 0.29	17.92 ± 0.023	0.498 ± 0.022	32.73 ± 0.027
4	23.11 ± 0.28	103.37 ± 0.25	205.33 ± 0.27	11.67 ± 0.091	0.468 ± 0.033	28.71 ± 0.515
5	23.76 ± 0.26	110.54 ± 0.27	225.42 ± 0.31	13.77 ± 0.037	0.462 ± 0.005	30.88 ± 0.347
6	27.32 ± 0.31	117.63 ± 0.25	221.15 ± 0.34	13.45 ± 0.015	0.462 ± 0.038	31.14 ± 0.342
7	36.15 ± 0.19	122.71 ± 0.29	227.71 ± 0.38	8.66 ± 0.051	0.449 ± 0.004	26.54 ± 0.263
8	42.21 ± 0.26	129.22 ± 0.38	235.86 ± 0.39	7.84 ± 0.028	0.446 ± 0.045	25.79 ± 0.532
9	112.23 ± 0.31	142.13 ± 0.35	256.15 ± 0.34	8.21 ± 0.024	0.443 ± 0.009	26.12 ± 0.295

**Table 6:** Regression analysis of granules measured responses.

Variables	Coefficient Estimate	Sum of Squares	Standard Error	F-Value	*p*-Value	95 % CI Low	95 % CI High
**Y_1_: D50 (Quadratic model)**							
**Intercept**	109.38	-	0.8256	-	-	106.75	112.01
**X_1_**	36.37	7935.93	0.4522	6468.77	**<0.0001**	34.93	37.81
**X_2_**	7.33	322.52	0.4522	262.89	**0.0005**	5.89	8.77
**X_1_ X_2_**	2.28	20.75	0.5538	16.91	**0.0260**	0.515	4.04
**X_1_^2^**	−15.53	482.26	0.7832	393.10	**0.0003**	−18.02	−13.04
**X_2_^2^**	1.7	5.79	0.7832	4.72	**0.1182**	−0.7908	4.19
**Y_2_: Percent fines (Linear model)**							
**Intercept**	13.11	-	0.2298	-	-	12.54	13.67
**X_1_**	−4.94	146.42	0.2815	308.04	**<0.0001**	−5.63	−4.25
**X_2_**	0.19	0.21	0.2815	0.45	**0.5248**	−0.49	0.87
**Y** **_3_** **: Bulk density (Quadratic model)**							
**Intercept**	0.463	-	0.0012	-	-	0.455	0.4715
**X_1_**	−0.032	0.0063	0.0014	539.80	**0.0002**	−0.0368	−0.0279
**X_2_**	−0.006	0.0002	0.0014	19.64	**0.0214**	−0.0106	−0.0017
**X_1_ X_2_**	0.004	0.0001	0.0017	7.77	**0.0686**	−0.0007	0.0102
**X_1_^2^**	0.0143	0.0004	0.0024	35.36	**0.0095**	0.0067	0.0220
**X_2_^2^**	0.0008	1.389 × 10^−6^	1.389 × 10^−6^	0.1195	**0.7524**	−0.0068	0.0085
**Y** **_4_** **: Angle of repose (Linear model)**							
**Intercept**	29.77	-	0.2842	-	-	29.07	30.46
**X_1_**	−3.38	68.41	0.3481	94.12	**<0.0001**	−4.23	−2.52
**X_2_**	0.31	0.5828	0.3481	0.80	**0.4050**	−0.54	1.16

X_1_ and X_2_ are water amount and spray rate, respectively; X_1_X_2_ is the interaction effect.

**Table 7:** Measured response results of prepared tablets (mean ± SD).

Formula	Weight (mg ± SD)	Thickness (mm ± SD)	Tensile Strength (MPa ± SD)	Friability (% ± SD)	Disintegration Time (s ± SD)	%release at 30 min (% ± SD)
1	497.56 ± 1.35	4.12 ± 0.011	2.53 ± 0.64	1.22 ± 0.02	34.25 ± 2.13	73.23 ± 3.15
2	498.93 ± 1.37	4.31 ± 0.004	2.51 ± 0.13	1.24 ± 0.02	31.69 ± 1.31	75.15 ± 1.14
3	501.71 ± 1.33	4.33 ± 0.006	2.73 ± 0.65	1.19 ± 0.02	44.91 ± 1.26	76.66 ± 2.13
4	499.78 ± 1.03	4.23 ± 0.02	2.89 ± 0.38	0.97 ± 0.02	51.36 ± 1.67	81.25 ± 2.35
5	498.84 ± 1.18	4.12 ± 0.003	2.86 ± 0.58	0.94 ± 0.04	53.21 ± 1.41	85.11 ± 1.84
6	497.75 ± 1.21	4.31 ± 0.007	2.88 ± 0.91	0.96 ± 0.05	56.33 ± 1.34	89.45 ± 2.48
7	498.45 ± 0.88	4.32 ± 0.004	3.11 ± 0.61	0.76 ± 0.01	131.67 ± 2.19	86.76 ± 2.81
8	498.63 ± 0.73	4.41 ± 0.001	3.15 ± 0.29	0.73 ± 0.02	138.57 ± 1.57	89.32 ± 2.21
9	501.23 ± 0.78	4.33 ± 0.03	3.23 ± 0.24	0.71 ± 0.01	143.12 ± 0.86	90.07 ± 3.17

**Table 8:** Regression analysis of tablets measured responses.

Variables	Coefficient Estimate	Sum of Squares	Standard Error	F-Value	*p*-Value	95 % CI Low	95 % CI Low
**Y_5_:** **SD of weight variation (Linear model)**							
**Intercept**	1.10	-	0.027	-	-	1.03	1.16
**X_1_**	−0.27	0.4593	0.033	70.02	**0.0002**	−0.3576	−0.1958
**X_2_**	0.01	0.0160	0.033	0.09	**0.7725**	−0.0709	0.0909
**Y_6_:** **Tensile strength (Linear model )**							
**Intercept**	2.88	-	0.020	-	-	2.83	2.93
**X_1_**	0.28	0.4931	0.024	137.49	**<0.0001**	0.2268	0.3465
**X_2_**	0.05	1.05	0.024	4.47	**0.0790**	−0.0082	0.1115
**Y_7_:** **Friability (Linear model)**							
**Intercept**	0.96	-	0.006	-	-	0.9528	0.9849
**X_1_**	−0.24	0.3504	0.008	905.38	**<0.0001**	−0.2613	−0.2220
**X_2_**	−0.01	0.0014	0.008	3.49	**0.111**	−0.0347	0.0047
**Y_8_:** **Disintegration time (Quadratic model)**							
**Intercept**	52.00	-	2.82	-	-	43.02	60.98
**X_1_**	50.42	15252.05	1.55	1063.40	**<0.0001**	45.50	55.34
**X_2_**	4.51	122.22	1.55	8.52	**0.0615**	−0.407	9.43
**X_1_ X_2_**	0.19	0.15	1.89	0.01	**0.923**	−5.83	6.22
**X_1_^2^**	33.73	2276.10	2.68	158.69	**0.0011**	25.21	42.26
**X_2_^2^**	2.45	12.00	2.68	0.8370	**0.4277**	−6.07	10.97
**Y_9_:** **Percent release at 30 min (linear model)**							
**Intercept**	83.00	-	0.7647	-	**-**	81.13	84.87
**X_1_**	6.85	281.67	0.9366	53.52	**0.0003**	4.56	9.14
**X_2_**	2.49	37.20	0.9366	7.07	**0.0376**	0.1982	4.78

X_1_ and X_2_ represent the amount of water and the spray rate, X_1_X_2_ is the effect of interaction and X_1_^2^& X_2_^2^ are the sum of effects.

**Table 9:** Similarity factor (f_2_) of dissolution profiles of different runs.

Comparison	F_2_ Value	Dissolution Profile
Run 1 # 4	59	Similar
Run 1 # 7	49	Disimilar
Run 2 # 5	54	Similar
Run 2 # 8	46	Disimilar
Run 3 # 6	54	Similar
Run 3 # 9	46	Disimilar
Run 4 # 7	70	Similar
Run 5 # 8	70	Similar
Run 6 # 9	62	Similar

**Table 10:** The constraints adopted for developed design space.

Variables	Target	Range	Weight	Importance Co-Efficient
**In-put**				
Water amount	In range	1–6% *w*/*w*	1	Not applicable
Spray rate	In range	2–8 g/min	1	Not applicable
**Out-put**				
SD of weight variation	0.8	0.73–1.37	1	+++
Tensile strength	Maximize	2.51–3.23 MPa	1	+++
Friability	0.75	0.71–1.24%	1	+++
Disintegration time	120	31.69–143.12 s	1	+++
Percent release after 30 min	85	73.23–90.07%	1	+++

**Table 11:** Predicted and experimental values of optimized run with their prediction errors.

**Variables**	**Value**		
Water amount (% *w*/*w*)	5.69		
Spray rate (g/min)	2.0		
Overall desirability = 0.876			
**Responses**	**Predicted Values**	Experimental Values *	**Prediction Error (%)**
SD of weight variation	0.84	0.82	2.38
Tensile strength (MPa)	3.07	3.20 ± 0.46	−4.23
Friability (%)	0.77	0.74 ± 0.05	3.89
Disintegration time (sec)	120	125.12 ± 2.43	−4.26
Percent release after 30 min (%)	86.52	88.21 ± 3.31	−1.95

* Experimental (actual) values are presented as mean ± SD.

## Data Availability

The datasets used and/or analyzed during the current study are available from the corresponding author on reasonable request.
